# Impact of surgeon and hospital factors on length of stay after colorectal surgery systematic review

**DOI:** 10.1093/bjsopen/zrac110

**Published:** 2022-09-19

**Authors:** Zubair Bayat, Keegan Guidolin, Basheer Elsolh, Charmaine De Castro, Erin Kennedy, Anand Govindarajan

**Affiliations:** Division of General Surgery, Department of Surgery, University of Toronto, Toronto, Ontario, Canada; Institute of Health Policy Management and Evaluation, University of Toronto, Toronto, Ontario, Canada; Sinai Health System, Toronto, Ontario, Canada; Division of General Surgery, Department of Surgery, University of Toronto, Toronto, Ontario, Canada; Division of General Surgery, Department of Surgery, University of Toronto, Toronto, Ontario, Canada; Sinai Health System, Toronto, Ontario, Canada; Division of General Surgery, Department of Surgery, University of Toronto, Toronto, Ontario, Canada; Institute of Health Policy Management and Evaluation, University of Toronto, Toronto, Ontario, Canada; Sinai Health System, Toronto, Ontario, Canada; Division of General Surgery, Department of Surgery, University of Toronto, Toronto, Ontario, Canada; Institute of Health Policy Management and Evaluation, University of Toronto, Toronto, Ontario, Canada; Sinai Health System, Toronto, Ontario, Canada

## Abstract

**Background:**

Although length of stay (LOS) after colorectal surgery (CRS) is associated with worse patient and system level outcomes, the impact of surgeon and hospital-level factors on LOS after CRS has not been well investigated. The aim of this study was to synthesize the evidence for the impact of surgeon and hospital-level factors on LOS after CRS.

**Methods:**

A comprehensive database search was conducted using terms related to LOS and CRS. Studies were included if they reported the effect of surgeon or hospital factors on LOS after elective CRS. The evidence for the effect of each surgeon and hospital factor on LOS was synthesized using vote counting by direction of effect, taking risk of bias into consideration.

**Results:**

A total of 13 946 unique titles and abstracts were screened, and 69 studies met the inclusion criteria. All studies were retrospective and assessed a total of eight factors. Surgeon factors such as increasing surgeon volume, colorectal surgical specialty, and progression along a learning curve were significantly associated with decreased LOS (effect seen in 87.5 per cent, 100 per cent, and 93.3 per cent of studies respectively). In contrast, hospital factors such as hospital volume and teaching hospital status were not significantly associated with LOS.

**Conclusion:**

Provider-related factors were found to be significantly associated with LOS after elective CRS. In particular, surgeon-related factors related to experience specifically impacted LOS, whereas hospital-related factors did not. Understanding the mechanisms underlying these relationships may allow for tailoring of interventions to reduce LOS.

## Teaser

We synthesized the evidence for the impact of surgeon and hospital factors on length of stay (LOS) in hospital after colorectal surgery (CRS). Provider-related factors, particularly surgeon factors, were found to impact LOS after elective CRS. Understanding the mechanisms underlying these relationships may allow for tailoring of interventions to reduce LOS after CRS.

## Introduction

Colorectal surgery (CRS) is performed in large numbers, across the world, to treat benign and malignant conditions. Due to the nature of CRS, most patients are admitted to hospital until they are able to take adequate nutrition and ambulate. During admission, patients are monitored for life-threatening complications, including bleeding and anastomotic leak.

Increased length of stay (LOS) is important because it has effects on both the healthcare system and patients. At a system level, increased LOS results in inpatient beds remaining occupied, leading to other surgeries being delayed and can lead to increases in healthcare costs. At a patient level, increased LOS has been associated with poorer patient outcomes, including thromboembolic disease^1–3^, iatrogenic complications^4–6^, deconditioning^7^, and nosocomial infections^8^. Therefore, decreasing LOS is critical to enhance patient outcomes and decrease healthcare utilization and costs.

LOS after CRS may be influenced by many factors, including overall health status of the patients, type of surgical procedure, elective, or emergency surgery, technical factors related to the surgery, and postoperative complications. Enhanced recovery after surgery (ERAS) programmes have also been shown to reduce LOS after CRS^9,10^, but how they are applied at the patient level is related to the provider. Therefore, examining how surgeon and hospital factors may impact LOS after CRS and the cumulative effects of these factors on LOS is important to further understand how LOS may be further reduced.

To date, while several studies have assessed surgeon and hospital factors, the results of these studies have been conflicting. Therefore, the objective of this study was to perform a systematic review to assess the body of existing evidence for the impact of surgeon and hospital factors on LOS after elective CRS.

## Methods

### Overview, search strategy, and study selection

PRISMA guidelines^11^ were followed throughout the search, review, reporting, and discussion (*Appendix S1*). The protocol for this review was registered prospectively with PROSPERO (CRD42020189058)^12^.

Online searches of the Ovid Cochrane Central Register of Controlled Trials, Ovid Cochrane Database of Systematic Reviews, Ovid Embase, and Ovid MEDLINE (supplemented by PubMed) were conducted, each from inception to 9 July 2020. Ongoing trials were searched in the US National Library of Medicine’s Clinicaltrials.gov database. Grey literature was searched using Google Scholar, where the first 20 pages of results were reviewed. The search terms and strategies employed for this review were designed with an information specialist (*Appendix S2*) and were peer-reviewed by a different information specialist (following PRESS guidelines)^13^. No age or language restrictions were applied to the literature searches. Duplicate studies were removed using EndNote X8 (Clarivate Analytics, Philadelphia, Pennsylvania, USA). The reference lists of included studies were hand-searched for additional relevant citations.

### Inclusion criteria

Studies were included if they assessed the effect of surgeon or hospital-level factors (factors inherent to the surgeon or the hospital at which the surgery was performed) on LOS after CRS. Studies that assessed the impact of laparoscopy or ERAS on LOS were not included unless they assessed the provider specifically. Randomized clinical trials (RCTs) and observational studies in which LOS was compared between groups (for example, cohort studies) were eligible for inclusion in the review. If systematic reviews assessing the impact of surgeon or hospital factors on LOS after CRS were found, studies within those reviews were assessed for inclusion in this review.

### Exclusion criteria

Exclusion criteria included: case series (in which no between-group comparisons were made); abstracts and conference proceedings^14^; studies performed before the year 2000; studies with fewer than 50 patients; and studies conducted on paediatric or emergency CRS populations.

Screening for inclusion/exclusion was performed using Distiller SR (Evidence Partners, Ottawa, Ontario, Canada). All titles and abstracts were screened, selecting only studies of surgeon and hospital-related factors. The inclusion and exclusion criteria were then applied to articles selected for full-text screening. All studies were screened by two independent reviewers (Z.B. and K.G./B.E.). Disagreements were resolved by discussion until consensus was achieved. When necessary, the reviewers contacted the study authors to obtain additional information.

### Risk of bias

Risk of bias (ROB) was assessed using the Newcastle–Ottawa scale^15^ for cohort studies, which graded studies on cohort selection, comparability, and outcome/exposure measures, and allowed for an overall estimation of study quality. Based on this scale, study quality was categorized as good, fair, or poor corresponding to low, moderate, or high ROB. ROB for RCTs was assessed using the Cochrane Collaboration’s Risk of Bias tool^16^. Cohort studies were classified and reported as good, fair, or poor quality and RCTs were classified low risk, some concerns, or high risk. ROB assessments were conducted by two independent reviewers (Z.B. and K.G./B.E.). Disagreements were resolved by discussion and a third independent reviewer was included if consensus could not be achieved.

### Data collection and synthesis

Data were abstracted from full texts using custom-built forms in Microsoft^®^ Word (Microsoft, Redmond, Washington, USA). The characteristics and results of each included study were extracted and tabulated. Data on the use of ERAS in the studies were extracted. The evidence for the independent impact of surgeon and hospital-related factors on LOS after CRS was synthesized if there were more than two studies assessing that factor, as these would represent clinically meaningful factors with sufficient evidence to synthesize. Studies were not included in the synthesis if the effect of the factor on LOS after CRS could not be determined (for example not reported or could not be extracted from tables). The evidence for each factor was presented in a tabular format.

Tabulated data were assessed qualitatively for heterogeneity by two reviewers, to determine whether patients, surgeries, exposure assessments, and outcome assessments were similar enough for meta-analysis to be performed. As described in the Cochrane Handbook for Systematic Reviews of Interventions, if heterogeneity in one or more of these domains precluded meta-analysis, evidence was synthesized using the vote-counting method^17^. This method allows for the direction of effect of a factor on LOS to be determined as well as whether the direction of effect is statistically significant. The number of studies for each factor that demonstrated a positive association with LOS and the number showing a negative association with LOS were enumerated. Results are reported as the proportion of studies showing an association in a given direction relative to the total number of studies, with 95 per cent Wilson confidence intervals^18^. The binomial test was used to test the hypothesis that the proportion was significantly different from 50 per cent, with a two-sided *P* value of 0.05 as the level of significance. If the Cochrane vote-counting proportion was significantly different from 50 per cent, one can infer that the factor was significantly associated with LOS. Binomial tests were not performed for observed proportions of 0 or 100 per cent, as the test performs poorly for observed probabilities approaching 0 or 100 per cent^18^.

Sensitivity analyses were performed, including only good-quality studies (low ROB) to assess the robustness of the results to potentially biased studies. The overall certainty of the evidence for each factor was assessed by examining the proportion of studies at high ROB, the precision around effect estimates and the consistency of study effects. Based on these criteria, the certainty in the overall effect for each factor was categorized as high, medium, low, or very low. This approach has been demonstrated to be effective and reproducible^19^. If no determination about the overall effect of a determinant on LOS could be made, then the certainty in the overall effect was not estimated.

## Results

**Fig. 1:**
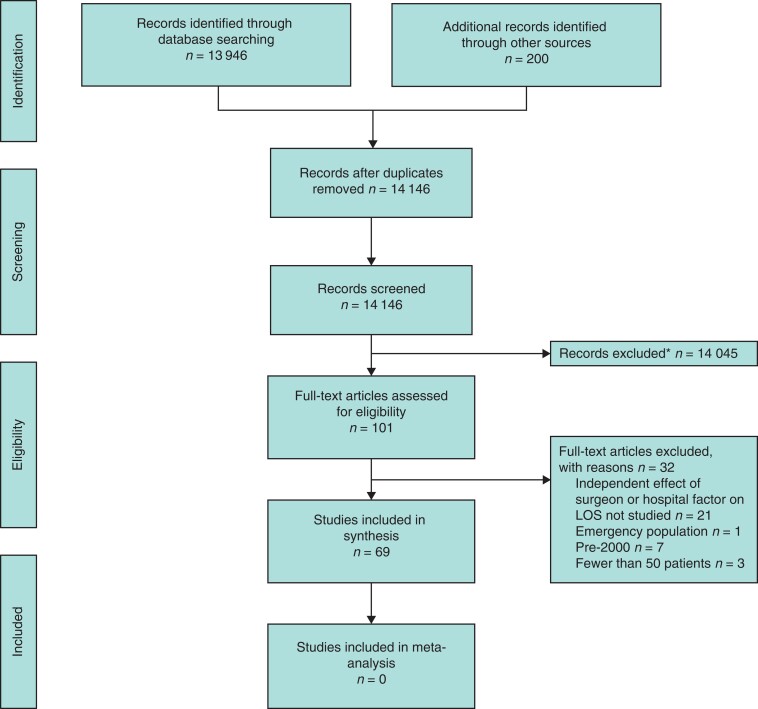
PRISMA flow diagram *Records were excluded if an independent effect of surgeon or hospital factor on LOS after colorectal surgery was not studied, if records were identified as duplicates missed by deduplication software, if records were identified as conference abstracts (*n* = 42), or if full texts could not be obtained (*n* = 2). Figure was adapted from Moher *et al*.^11^. For more information, see www.prisma-statement.org. LOS, length of stay.

The search strategy returned 18 918 citations and, after the removal of 5172 duplicate citations, 13 946 titles and abstracts were screened. The grey literature search returned 200 citations, of which 10 met criteria for full-text review. Of these, three were found to be duplicates and three further studies met the criteria for inclusion in the review (*Fig. 1*). In total, 101 full texts were reviewed, from which 32 were excluded, resulting in 69 studies for analysis. All included studies were retrospective cohort studies and the median study size was 1241 patients. Forty-one per cent (28 of 69) of the included studies were of good quality, whereas 1 per cent and 58 per cent of studies were fair or poor quality respectively.

Of the 69 studies included in this review, three commented on whether ERAS protocols were followed as part of the study^20–22^. The majority of studies included in this review were conducted using health administrative data or other similar data sources and therefore data about the specific elements of the ERAS protocols, uptake, compliance, and fidelity could not be obtained.

Twelve surgeon and hospital factors were reported in the included studies. Of the 12 factors, eight factors (four surgeon-level and four hospital-level) were reported in more than two studies and were included in the review. These studies are shown in *Table 1*. The remaining four factors were reported in two or fewer studies and were not included in the review (*Appendix S3*). In general, the included studies were performed on patient populations with varying proportions of patients with benign and malignant disease, undergoing many different types of CRS procedures and used differing exposure and outcome definitions. This heterogeneity between these studies precluded meta-analysis.

**Table 1:** Summary of review findings

Prolonged LOS with	Number of studies synthesized	Proportion (%) showing effect (95% c.i.)	Certainty in effect
Low-volume surgeons	16	87.5 (64–97)	High
Non-specialist surgeons	6	100 (65–100)	High
Early-learning curve	15	93.3 (70–99)	Moderate
More trainee involvement	13	61.5 (36–82)	Low
Low hospital volume	13	69.2 (42–87)	Low
Teaching hospitals	8	62.5 (31–86)	Low
Urban hospitals	3	66.7 (21–94)	Very low
Private hospitals	4	75 (30–95)	Very low

LOS, length of stay.

### Surgeon volume

Eighteen studies investigating the effect of surgeon volume on LOS after CRS were included^23–40^ (*Table S1*). In two studies^33,36^, the direction of the association between surgeon volume and LOS after CRS could not be determined, therefore 16 studies were synthesized. The included studies had sample sizes between 957 and 113 633 patients (median 6033 patients) and comprised patients undergoing laparoscopic and open surgery for benign and malignant diagnoses. These studies used various definitions of high and low volume (range 2–30 cases per year) and therefore a consistent threshold could not be determined.

Overall, there was a statistically significant association between increasing surgeon volume and decreased LOS with 14 of 16 studies showing such an association (Cochrane vote-counting proportion 87.5 per cent, 95 per cent c.i. 64 to 97 per cent, *P* = 0.004). Of these studies, 10 were of good quality (low ROB), one was of fair quality (moderate ROB), and five were of poor quality (high ROB). Sensitivity analysis including only good-quality studies did not alter the findings with 10 of 10 studies (100 per cent) demonstrating an association between increasing surgeon volume and decreased LOS. Overall, the level of certainty in this effect is high.

### Surgeon specialty

Six studies assessing the impact of surgeon specialty on LOS after CRS were included (*Table S2*)^23,29,32,41–43^. Studies ranged in size from 1190 to 270 648 patients (median 11 473 patients) and comprised patients undergoing CRS for benign and malignant diagnoses. ‘CRS specialists’ were defined in different ways that included surgeons with CRS fellowship training^36,42^, surgeons performing restorative proctectomy^43^, surgeons with CRS comprising more than 75 per cent of their case volume^29^, and surgeons self-reporting a CRS specialty.

CRS specialty was associated with decreased LOS after CRS in all studies (Cochrane vote-counting proportion 100 per cent). Two studies were of poor quality (high ROB) due to unadjusted analyses or inadequately described multivariable regression^42,43^, while the remainder were of good quality. Sensitivity analysis including only good-quality studies showed the same result. Overall, the level of certainty in this finding is high.

### Learning curve

Eighteen studies assessing the impact of a surgeon’s ‘learning curve’ on LOS after CRS were included^20,21,44–59^ (*Table S3*). The direction of this effect was not determinable in three of these studies^21,55,57^, and therefore 15 studies were synthesized. The included studies ranged from 66 to 31 709 patients (median 121 patients) and included patients undergoing a wide range of CRS (colectomy, proctectomy, robotic CRS, laparoscopic CRS, and transanal total mesorectal excision) for benign and malignant indications. Two broad definitions of learning curve were used, with some studies defining the learning curve as the first portion of a surgeon’s cases (first eighth, quarter, third, or half of the surgeon’s cases during the study interval) and others using the cumulative sum (CUSUM) method^47^. None of the studies reported whether the surgical procedure was standardized or whether the quality of the surgery was assessed.

In 14 of 15 synthesized studies, CRS early in the learning curve was associated with increased LOS (Cochrane vote-counting proportion 93.3 per cent (95 per cent c.i. 70 to 99 per cent), *P* < 0.001). Fourteen of the included studies were of poor quality (high ROB) due to their unadjusted analyses. Only the study by Symer *et al.*^44^ was of good quality (low ROB) and demonstrated increased LOS early in the learning curve. Given the consistency of the findings in the included studies but the paucity of studies at low ROB, there is moderate certainty in the association between CRS early in the learning curve and increased LOS after CRS.

### Trainee involvement

Fifteen studies evaluating the impact of trainee involvement on LOS after CRS were included^22,36,60–72^ (*Table S4*). In two studies^36,66^, the direction of this effect could not be determined and 13 studies were synthesized. Study sample sizes ranged between 78 and 7254 patients (median 451 patients). Studies included CRS performed for benign and malignant indications—6 of the 13 studies were limited to laparoscopic CRS^22,60,61,64,65,71^, while the remaining seven studies included laparoscopic and open CRS. Four studies compared cases with resident participation to those with none^60,68,70,72^, whereas the remaining nine studies compared surgeries with differing degrees of trainee involvement (for example trainee assistant *versus* main operator).

Overall, there was no statistically significant association between increasing trainee involvement and LOS after CRS with 8 of the 13 synthesized studies^22,60,61,63,65,67,70,71^, showing an association between trainee involvement and increased LOS after CRS (Cochrane vote-counting proportion 61.5 per cent (95 per cent c.i. 36 to 82 per cent), *P* = 0.581). Five of the six studies limited to laparoscopic CRS demonstrated an association between increasing trainee involvement and increased LOS after CRS, while only three of seven studies including all CRS demonstrated this effect. Four of the 13 studies included were of good quality (low ROB), while the remainder were of poor quality (high ROB). Sensitivity analysis including only good-quality studies also showed no statistically significant association, with three of the four good-quality studies demonstrating an association between increasing trainee involvement and LOS after CRS (Cochrane vote-counting proportion 75 per cent (95 per cent c.i. 30 to 95 per cent), *P* = 0.625).

### Hospital volume

Seventeen studies of the relationship between hospital volume and LOS after CRS were included^25,26,28,30,33,37–39,41,73–80^ (*Table S5*). In four studies, the direction of the association between hospital volume and LOS could not be determined^38,73,76,78^, and these studies were not synthesized. The 13 synthesized studies included between 536 and 186 013 patients (median 9306 patients), undergoing diverse CRS for benign and malignant conditions. The thresholds for hospital volume varied between studies, ranging between 3.3 and 110 cases per year. Studies used heterogeneous thresholds for high-volume hospitals.

Overall, there was no statistically significant association between hospital volume and LOS after CRS. Nine of 13 (69 per cent) studies showed an association between increasing hospital volume and decreasing LOS with four studies showing the opposite effect^25,26,39,79^ (Cochrane vote-counting proportion 69.2 per cent (95 per cent c.i. 42 to 87 per cent), *P* = 0.267). Nine of the 13 included studies were of good quality (low ROB), one was of fair quality (moderate ROB), and three were of poor quality (high ROB). Sensitivity analysis including only the nine good-quality studies revealed a similar proportion of studies demonstrating an association between increasing hospital volume and decreased LOS after CRS (Cochrane vote-counting proportion 66.7 per cent, (95 per cent c.i. 35 to 88 per cent), *P* = 0.508).

### Teaching hospital

Eight studies assessing the impact of teaching hospitals on LOS after CRS were included^29,41,78,81–85^ (*Table S6*). These studies included between 3765 and 115 250 patients (median 22 625 patients), treated with CRS for benign and malignant indications. The definition of teaching hospitals varied between studies and included teaching hospitals as hospitals affiliated with medical schools^41,82,84^, but also accepted hospitals with general surgery residency programmes^82^, National Cancer Institute Comprehensive Cancer Care Programs^84^, or hospitals with a Dean’s Committee and a general surgery resident as teaching hospitals^85^.

Overall, there was no statistically significant association between teaching hospital status and LOS after CRS. Five of eight (63 per cent) of the included studies demonstrated an association between CRS at teaching hospitals and increased LOS after CRS^29,81–83,85^ (Cochrane vote-counting proportion 62.5 per cent (95 per cent c.i. 31 to 86 per cent), *P* = 0.727). Notably, the five studies demonstrating an association between teaching hospitals and increased LOS included laparoscopic and open CRS, while the three studies demonstrating an association between teaching hospitals and decreased LOS included only laparoscopic CRS^41,78,84^. Of the eight included studies, six were of good quality (low ROB) and two were of poor quality (high ROB). Sensitivity analysis including only good-quality studies resulted in a similar proportion of studies (4 of 6) demonstrating an association between teaching hospitals and increased LOS (Cochrane vote-counting proportion 66.7 per cent (95 per cent c.i. 30 to 90 per cent), *P* = 0.688). Given that the overall effect was non-significant and that there was limited consistency between studies, the effect of teaching hospital on LOS after CRS is unclear.

### Other factors: rural hospital and private hospital ownership

Three studies assessing the impact of rural hospitals on LOS after CRS were identified^24,86,87^, (*Table S7*) as were five studies assessing the effect of private hospital ownership on LOS after CRS^25,41,72,73,88^ (*Table S8*). There was no statistically significant association between rural hospital status or private hospital ownership and LOS after CRS (Cochrane vote-counting proportion 66.7 per cent (95 per cent c.i. 21 to 94 per cent), *P* > 0.999 for rural hospitals and Cochrane vote-counting proportion 75 per cent (95 per cent c.i. 30 to 95 per cent), *P* = 0.625 for private hospitals). Sensitivity analysis including only studies at low ROB showed similar results. Given the small number of studies on these factors and the overall poor quality of the evidence, the overall certainty in these effects is very low.

## Discussion

In this systematic review, the impact of surgeon and hospital-based factors on LOS after CRS was evaluated. The results supported an association between surgeon factors but not hospital factors on LOS. Surgeon factors that were associated with LOS with a high level of certainty were surgeon volume, surgeon specialty, and learning curve.

The impact of surgeon factors on LOS may be mediated through differences in the use of ERAS protocols, which can be associated with reduced LOS^9,10^. ERAS protocols are generally put in place at the hospital or a department level, but compliance is determined by individual patients and surgeons. Therefore, the finding that surgeon factors but not hospital factors impact LOS may suggest that surgeon volume and specialty may be associated with the fidelity and consistency with which ERAS protocols are applied to patients. In this review, only three studies reported the use of and compliance with an ERAS protocol, and thus it was not possible to elicit the extent to which provider variation in the use of ERAS played a role in LOS.

While it is possible that higher volume and specialist surgeons more readily implement ERAS protocols, it is unlikely that implementation of ERAS into their practice is the sole reason for decreased LOS. More experienced or subspecialty surgeons may also perform more minimally invasive surgeries or higher-quality surgeries^89^ that result in a broad array of improved outcomes, including fewer complications and better rescue from complications that lead to a decrease in LOS^90–92^. In general, the literature did not adequately control for such possible mediating and confounding factors, and therefore it was not possible to assess the influence of this on the study findings. Specifically, none of the studies addressed the quality or standardization of the surgeries performed.

The impact of the surgeon experience and training on LOS may also be related to behavioural differences among surgeons with respect to the subjective threshold for discharge^91^. An example of this would be the practice of same-day or short-stay colectomy by some providers, or the differences in mean LOS in different countries^93^. Such behavioural differences would represent a discretionary impact on LOS that may be modifiable through implementation of protocolized postoperative care pathways. Surgeon behaviours may also be influenced by cognitive or recall biases related to previous cases, as well as the culture of their surgical colleagues^94,95^ and the hospital. The true effects of hospital-related factors on LOS after CRS may also not captured in the existing literature because of small effect sizes that may be diluted or mediated by surgeon-related factors^96,97^, as the surgeons directly guide patient care.

This is the first synthesis of the evidence in the literature focusing on the impact of providers themselves on LOS. To ensure that the results were relevant to contemporary colorectal practice, the review was restricted to studies published after the year 2000, a time interval when ERAS had already been established. Although a broad and inclusive search strategy was used, only eight factors that were assessed by multiple studies were identified. In addition, the studies were of variable quality and did not use consistent definitions for factors such as volume and learning curve, precluding the ability to perform a meta-analysis of the results and derive evidence-based thresholds for these factors. To mitigate against the quality of the included studies, pre-planned sensitivity analyses restricted to only good-quality studies at low ROB were incorporated. The existing literature also did not elucidate potential mechanisms by which surgeons with more training and experience are associated with reduced LOS. For example, most studies did not describe the degree to which ERAS protocols were utilized. Understanding these mechanisms are an important step towards devising interventions to translate these improvements into practice.

Surgery by high-volume surgeons, by specialist surgeons, and by surgeons that have progressed past their initial learning curve was associated with shorter LOS after CRS. In contrast, hospital factors did not seem to have a consistent impact on LOS. The literature, however, has significant gaps. Given that CRS is widely performed, further study and evidence-based actions to reduce LOS (such as ERAS protocols and standardization of care) can result in large cumulative improvements in LOS, benefitting patients and the healthcare system as a whole.

## Supplementary Material

zrac110_Supplementary_Data

## Data Availability

Data are available upon request from the corresponding author. Study registration already in the Methods section (which is the correct place to include it).
