# Supplementary material for: Impact of surgeon and hospital factors on length of stay after colorectal surgery systematic review
Source: BJS Open. 2022 Sep 19;6(5):zrac110. doi: 10.1093/bjsopen/zrac110 (PMC9487584; doi:10.1093/bjsopen/zrac110)
Supplement: zrac110_Supplementary_Data [file zrac110_supplementary_data.zip › Supplementary_Appendix_2.docx]

**Appendix S2: Search Strategies**

MEDLINE Search:

Database(s): Ovid MEDLINE(R) ALL <1946 to July 07, 2020>
Search Strategy:

| **#** | **Searches** |
| --- | --- |
| 1 | Length of Stay/ |
| 2 | hospitalization/ |
| 3 | Patient Discharge/ |
| 4 | Patient Readmission/ |
| 5 | (length? adj4 stay).tw,kf. |
| 6 | (hospital adj3 stay?).tw,kf. |
| 7 | (length? adj3 hospital*).tw,kf. |
| 8 | (time adj2 discharg*).tw,kf. |
| 9 | prolonged los.tw,kf. |
| 10 | long los.tw,kf. |
| 11 | excess los.tw,kf. |
| 12 | extended los.tw,kf. |
| 13 | or/1-12 |
| 14 | Colectomy/ |
| 15 | proctocolectomy, restorative/ |
| 16 | proctectomy/ |
| 17 | Colon/su [Surgery] |
| 18 | Rectum/su [Surgery] |
| 19 | colectom*.tw,kf. |
| 20 | (hemicolectom* or hemi-colectom*).tw,kf. |
| 21 | (proctocolectom* or procto-colectom* or proctectom* or coloproctocolectom*).tw,kf. |
| 22 | ((abdominoperineal or abdomino-perineal or large bowel or large intestin*) adj2 (excision? or resect* or remov*)).tw,kf. |
| 23 | ((rectum or rectal or colorectal or colon) adj2 (excision? or resect* or remov*)).tw,kf. |
| 24 | or/14-23 |
| 25 | 13 and 24 |

PUBMED Search (not including Medline):

PubMed (not including Medline)

**((((((((("Length of Stay"[Mesh]) OR "Hospitalization"[Mesh]) OR "Patient Discharge"[Mesh]) OR "Patient Readmission"[Mesh])) OR (((((((((length of stay[Title/Abstract]) OR hospital stay[Title/Abstract]) OR length in hospital[Title/Abstract]) OR time in hospital[Title/Abstract]) OR time to discharge[Title/Abstract]) OR prolonged los[Title/Abstract]) OR long los[Title/Abstract]) OR excess los[Title/Abstract]) OR extended los[Title/Abstract]))) AND ((((((((("Colectomy"[Mesh]) OR "Proctocolectomy, Restorative"[Mesh]) OR "Proctectomy"[Mesh]) OR "Colon/surgery"[Mesh]) OR "Rectum/surgery"[Mesh])) OR (((((((colectom*[Title/Abstract]) OR hemicolectom*[Title/Abstract]) OR hemi-colectom*[Title/Abstract]) OR proctocolectom*[Title/Abstract]) OR procto-colectom*[Title/Abstract]) OR proctectom*[Title/Abstract]) OR coloproctocolectom*[Title/Abstract])) OR (((abominoperineal[Title/Abstract] OR abdomino-perineal[Title/Abstract] OR large bowel[Title/Abstract] OR large intestine[Title/Abstract])) AND (excision[Title/Abstract] OR resection[Title/Abstract] OR removal[Title/Abstract]))) OR (((rectum[Title/Abstract] OR rectal[Title/Abstract] OR colorectal[Title/Abstract] OR colon[Title/Abstract])) AND (excision[Title/Abstract] OR resection removal[Title/Abstract])))))) AND pubmednotmedline [sb]**

EMBASE Search:

Database(s): OVID Embase Classic+Embase <1947 to 2020 July 08>
Search Strategy:

| **#** | **Searches** |
| --- | --- |
| 1 | Length of Stay/ |
| 2 | hospitalization/ |
| 3 | hospital discharge/ |
| 4 | hospital readmission/ |
| 5 | (length? adj4 stay).tw,kw. |
| 6 | (hospital adj3 stay?).tw,kw. |
| 7 | (length? adj3 hospital*).tw,kw. |
| 8 | (time adj2 discharg*).tw,kw. |
| 9 | prolonged los.tw,kw. |
| 10 | long los.tw,kw. |
| 11 | excess los.tw,kw. |
| 12 | extended los.tw,kw. |
| 13 | or/1-12 |
| 14 | colon resection/ |
| 15 | proctocolectomy/ |
| 16 | rectum resection/ |
| 17 | Colon/su [Surgery] |
| 18 | Rectum/su [Surgery] |
| 19 | colectom*.tw,kw. |
| 20 | (hemicolectom* or hemi-colectom*).tw,kw. |
| 21 | (proctocolectom* or procto-colectom* or proctectom* or coloproctocolectom*).tw,kw. |
| 22 | ((abdominoperineal or abdomino-perineal or large bowel or large intestin*) adj2 (excision? or resect* or remov*)).tw,kw. |
| 23 | ((rectum or rectal or colorectal or colon) adj2 (excision? or resect* or remov*)).tw,kw. |
| 24 | or/14-23 |
| 25 | 13 and 24 |

COCHRANE SR Search:

Database(s): EBM Reviews - Cochrane Database of Systematic Reviews on OVID <2005 to July 02, 2020>
Search Strategy:

| **#** | **Searches** |
| --- | --- |
| 1 | (length? adj4 stay).ti,ab,kw. |
| 2 | (hospital adj3 stay?).ti,ab,kw. |
| 3 | (length? adj3 hospital*).ti,ab,kw. |
| 4 | (time adj2 discharg*).ti,ab,kw. |
| 5 | LOS.ti,ab,kw. |
| 6 | 1 or 2 or 3 or 4 or 5 |
| 7 | colectom*.ti,ab,kw. |
| 8 | (hemicolectom* or hemi-colectom*).ti,ab,kw. |
| 9 | (proctocolectom* or procto-colectom* or proctectom* or coloproctocolectom*).ti,ab,kw. |
| 10 | ((abdominoperineal or abdomino-perineal or large bowel or large intestin*) adj2 (excision? or resect* or remov*)).ti,ab,kw. |
| 11 | ((rectum or rectal or colorectal or colon) adj2 (excision? or resect* or remov*)).ti,ab,kw. |
| 12 | 7 or 8 or 9 or 10 or 11 |
| 13 | 6 and 12 |

COCHRANE CENTRAL Search:

Database(s): EBM Reviews - Cochrane Central Register of Controlled Trials on OVID <June 2020>
Search Strategy:

| **#** | **Searches** |
| --- | --- |
| 1 | Length of Stay/ |
| 2 | hospitalization/ |
| 3 | Patient Discharge/ |
| 4 | Patient Readmission/ |
| 5 | (length? adj4 stay).tw,kw. |
| 6 | (hospital adj3 stay?).tw,kw. |
| 7 | (length? adj3 hospital*).tw,kw. |
| 8 | (time adj2 discharg*).tw,kw. |
| 9 | prolonged los.tw,kw. |
| 10 | long los.tw,kw. |
| 11 | excess los.tw,kw. |
| 12 | extended los.tw,kw. |
| 13 | or/1-12 |
| 14 | Colectomy/ |
| 15 | proctocolectomy, restorative/ |
| 16 | proctectomy/ |
| 17 | Colon/su [Surgery] |
| 18 | Rectum/su [Surgery] |
| 19 | colectom*.tw,kw. |
| 20 | (hemicolectom* or hemi-colectom*).tw,kw. |
| 21 | (proctocolectom* or procto-colectom* or proctectom* or coloproctocolectom*).tw,kw. |
| 22 | ((abdominoperineal or abdomino-perineal or large bowel or large intestin*) adj2 (excision? or resect* or remov*)).tw,kw. |
| 23 | ((rectum or rectal or colorectal or colon) adj2 (excision? or resect* or remov*)).tw,kw. |
| 24 | or/14-23 |
| 25 | 13 and 24 |
