# Supplementary material for: Impact of surgeon and hospital factors on length of stay after colorectal surgery systematic review
Source: BJS Open. 2022 Sep 19;6(5):zrac110. doi: 10.1093/bjsopen/zrac110 (PMC9487584; doi:10.1093/bjsopen/zrac110)
Supplement: zrac110_Supplementary_Data [file zrac110_supplementary_data.zip › Supplementary_Appendix_3.docx]

**Appendix S3: Surgeon and Hospital Determinants with Insufficient Evidence for Synthesis**

| **Determinants not synthesized** | **Titles and Abstracts Reviewed** | **Eligible Full Texts** |
| --- | --- | --- |
| Hospital Region | 6 | 1^1^ |
| Hospital Specialization | 3 | 1^2^ |
| Hospital Size | 3 | 2^1,3^ |
| Surgeon Years in Practice | 3 | 2^4,5^ |

References

1. Wilson MZ, Soybel DI, Hollenbeak CS. Operative volume in colon surgery: a matched cohort analysis. *Am J Med Qual Off J Am Coll Med Qual*. 2015;30:271–282.

2. Pandit V, Khalil M, Joseph B, Jandova J, Jokar TO, Haider AA, Zangbar B, Asim A, Hassan A, Nfonsam V. Disparities in Mangement of Patients with Benign Colorectal Disease: Impact of Urbanization and Specialized Care. *Am Surg*. 2016;82:1046–1051.

3. Altieri MS, Yang J, Telem DA, Chen H, Talamini M, Pryor A. Robotic-assisted outcomes are not tied to surgeon volume and experience. *Surg Endosc*. 2016;30:2825–2833.

4. Liu C-J, Chou Y-J, Teng C-J, Lin C-C, Lee Y-T, Hu Y-W, Yeh C-M, Chen T-J, Huang N. Association of surgeon volume and hospital volume with the outcome of patients receiving definitive surgery for colorectal cancer: A nationwide population-based study. *Cancer*. 2015;121:2782–2790.

5. Prystowsky JB, Bordage G, Feinglass JM. Patient outcomes for segmental colon resection according to surgeon’s training, certification, and experience. *Surgery*. 2002;132:663–670; discussion 670-672.
