# Supplementary material for: Impact of surgeon and hospital factors on length of stay after colorectal surgery systematic review
Source: BJS Open. 2022 Sep 19;6(5):zrac110. doi: 10.1093/bjsopen/zrac110 (PMC9487584; doi:10.1093/bjsopen/zrac110)
Supplement: zrac110_Supplementary_Data [file zrac110_supplementary_data.zip › Supplementary_Tables.docx]

**Table S1: Results of studies evaluating the impact of *surgeon volume* on LOS after CRS**

| **Authors, Country, Year** | **Number of Patients** | **Colorectal Surgeries Included** | **Exposure Definition** | **Impact on LOS** | **Effect Estimate** | **ERAS** | **Risk of Bias/**  **Quality (NOS)** |
| --- | --- | --- | --- | --- | --- | --- | --- |
| Yi et al, USA, 2018^24^ | 1190 | Colon and rectal resections | Highest quartile of surgeons by volume, compared to second and lowest two quartiles | Decreased | RC 0.8 for LOS after surgery by low compared to high volume surgeons. No significant effect between highest and 2^nd^ highest quartiles. p=0.008 | Not reported | Good |
| Aquina et al, USA, 2016^12^ | 6295 | Reversal of Hartmann’s colostomy | Lowest tertile of surgeons for volume during the study period | Decreased | Adjusted incident RR 0.87 for highest tertile of surgeons. No significant association with middle tertile. p-value not reported, significant per author. | Not reported | Good |
| Liu et al, Taiwan, 2015^11^ | 61,728 | Colorectal cancer surgeries | Lowest quartile of surgeons for CRS volume | Decreased | OR for pLOS 0.40, 0.58, 0.47 for highest, and middle quartiles. p<0.001 | Not reported | Good |
| Damle et al, USA, 2014^14^ | 17,749 | Colorectal cancer surgeries | Lowest 50% of surgeons for CRS volume | Decreased | RC -0.05 and -0.01 for medium and high-volume surgeons respectively. p-value not reported, not significant. | Not reported | Good |
| Burns et al, UK, 2014^25^ | 6554 | Laparoscopic colon cancer resection | High volume surgeons (highest tertile and highest 5%) | Decreased | Lowest surgeon volume tertile associated with longer LOS than medium and highest tertiles (RR 0.94 and 0.88 respectively). Low vs. medium p=0.001, low vs. high p<0.001 | Not reported | Good |
| Burns et al, UK, 2013^13^ | 109,261 | Colorectal cancer surgeries | Lowest tertile of surgeons for volume AND surgeon volume as a continuous variable | Decreased | OR 0.95 for pLOS for highest compared to lowest tertile of surgeons. p-value not reported, significant. No significant association when middle and lowest tertiles compared | Not reported | Good |
| Rea et al, USA, 2011^26^ | 113,633 | Colon and rectal resections | Surgeon volume (number of colorectal cases in study period). Continuous, no comparator | Decreased | Each additional annual colorectal case associated with decrease in LOS by 0.003 days. p=0.0119 | Not reported | Good |
| Burns et al, UK, 2011^27^ | 5771 | Restorative proctocolectomy (IPAA) | Surgeons in the lowest tertile for aggregated volume during the study period | Decreased | Highest tertile associated with shorter LOS than lowest tertile (RR 0.93). p<0.001 | Not reported | Good |
| Boudourakis et al, USA, 2009^28^ | 20867 | Colectomy | Lowest tertile of surgeons for colectomy volume compared to highest tertile | Decreased | RC for lowest tertile 1.1 (1999) and 0.8 (2005) at different time points in the study. p<0.001 (1999) and p<0.05 (2005) | Not reported | Good |
| Prystowski et al, USA, 2002^29^ | 15427 | Segmental colectomy | Surgeons performing more than 70 surgeries in 4 years | Decreased | RC -0.1 for high volume surgeons, compared to low volume surgeons. p-value not significant per author | Not reported | Good |
| Yasunaga et al, 2009^30^ | 1212 | Laparoscopic colon and rectal resections | Surgeons having performed fewer than 30 laparoscopic CRS, compared to those performing 30-99, 100-199, >200 laparoscopic CRS | Cannot determine | HR 1.13, 1.18, 0.97 for lowest quartile compared to second, third and highest quartiles respectively. p-values 0.19, 0.14, and 0.85 respectively | Not reported | Fair |
| Aleksic et al, Bosnia and Herzegovina, 2019^31^ | 546 | Anterior resection for rectal cancer | Surgeons performing fewer than 5 anterior resections per year, compared to surgeons performing 6-10 and >10 anterior resections per year | Increased | Mean LOS for lowest group 8.8 days, middle group 9.7 days and highest volume group 10.35 days. Lowest to middle group p=0.057. Lowest to highest volume group p<0.001. | Not reported | Poor |
| Bastawrous et al, USA, 2018 ^32^ | 957 | Robotic right and left colectomy, LAR, APR | High robotic CRS volume (more than 30 robotic CRS cases in index year) | Decreased | Mean LOS 1.6 days shorter for high volume surgeons. p<0.0001 | Not reported | Poor |
| Altieri et al, USA, 2016^33^ | 643 | Robotic colectomy | Lowest quartile of surgeons by volume compared to other volume quartiles | Cannot determine | No significant effect of surgeon volume on LOS after CRS. Parameter estimates not provided. p=0.0669 | Not reported | Poor |
| Keller et al, USA, 2013^34^ | 1428 | Robotic colectomy | Lowest tertile of surgeons for volume | Decreased | Mean LOS 2.8 days shorter for high volume surgeons. p<0.0001 | Not reported | Poor |
| Kelly et al, Ireland, 2012^35^ | 5133 | Colorectal cancer surgery | Lowest tertile of surgeons for colorectal cancer surgery volume during the study period | Decreased | OR for pLOS 0.76 and 0.83 respectively for middle and highest volume quartiles. p-value not reported, significant | Not reported | Poor |
| Drolet et al, Canada, 2011^36^ | 54000 | Colectomy for colon cancer | Lowest tertile of surgeons for CRS volume | Decreased | LOS for patients treated by medium tertile surgeons 4.9% shorter than for patients treated by lowest tertile. p-value not reported, significant per author. | Not reported | Poor |
| Larson et al, USA, 2008^37^ | 871 | Colectomy for colon cancer | Lowest tertile of credentialled surgeons for colectomy volume | Increased | Mean LOS 6.0 days for patients in lowest tertile and 6.1 days in each other tertile. p=0.95 | Not reported | Poor |

pLOS = prolonged LOS, RC = regression coefficient, OR = odds ratio, RR= risk ratio, HR = hazard ratio, ERAS = Study performed on patients enrolled in a fast-track surgery protocol, NOS = Newcastle Ottawa Scale

**Table S2: Results of studies evaluating the impact of *colorectal surgery specialty* on LOS after CRS**.

| **Authors, Country, Year** | **Number of Patients** | **Colorectal Surgeries Included** | **Exposure Definition** | **Impact on LOS** | **Effect Estimate** | **ERAS** | **Risk of Bias/ Quality (NOS)** |
| --- | --- | --- | --- | --- | --- | --- | --- |
| Yi et al, USA, 2018^24^ | 1190 | Colon and rectal resections | Cases by colorectal surgery specialists | Decreased | RC 0.2 for post-surgery LOS, p=0.364 ( -0.2, p=0.481 for overall LOS) after cases by general surgeons (compared to colorectal specialists). | Not reported | Good |
| Zheng et al, USA, 2014^38^ | 4617 | Laparoscopic colectomy for colon cancer | Colorectal specialty – Health Care Financing Administration | Decreased | Incident RR for LOS after surgery performed by CRS 0.89, compared to other surgeries. P-value not reported, significant. | Not reported | Good |
| Rea et al, USA, 2011^26^ | 113,633 | Colon and rectal resections | Cases by colorectal specialists (>75% caseload CRS) | Decreased | RC -0.3 for CRS specialists compared to other surgeons. p=0.022 | Not reported | Good |
| Prystowski et al, USA, 2002^29^ | 15427 | Segmental colectomy | Colorectal specialty – ABMS certification | Decreased | RC 1.0 for surgeons without CRS certification, compared to CRS. P<0.05 | Not reported | Good |
| Saraidaridis et al, USA, 2017^39^ | 270648 | Colon and rectal surgery | Cases by ABCRS-certified surgeons | Decreased | LOS 0.8 days shorter for patients treated by colorectal trained surgeons. p=0.001 | Not reported | Poor |
| Ricciardi et al, USA, 2011^40^ | 7519 | Proctectomy for rectal cancer | Cases by colorectal specialists (surgeons performing restorative proctectomy) | Decreased | LOS 9.2 days for patients treated by CRS specialists compared to 11.3 days for other surgeons. p=0.001 | Not reported | Poor |

pLOS = prolonged LOS, RC = regression coefficient, OR = odds ratio, RR= risk ratio, HR = hazard ratio, ERAS = Study performed on patients enrolled in a fast-track surgery protocol, NOS = Newcastle Ottawa Scale

**Table S3: Results of studies evaluating the impact of a *surgeon’s learning curve* on LOS after CRS**

| **Authors, Country, Year, RefID** | **Number of Patients** | **Colorectal Surgeries Included** | **Exposure Definition** | **Impact on LOS** | **Effect Estimate** | **ERAS** | **Risk of Bias/ Quality (NOS)** |
| --- | --- | --- | --- | --- | --- | --- | --- |
| Symer et al, USA, 2019^41^ | 2763 | Robotic colon and rectal cancer surgery | Cases performed in first quartile of learning curve (<9 surgeries) compared to other cases in cohort | Increased | 25.6% of cases with pLOS in earliest quartile decreasing to 12.8% in latest quartile. p<0.001 | Not reported | Good |
| Nasseri et al, USA, 2020^42^ | 111 | Robotic colon and rectal surgery | First 13 cases (CUSUM curve rising) to cases 14-83 and 84-111 (curve plateau and decreasing respectively) | Increased | Median LOS decreased from 6 to 3.5 days between earliest and latest group. p=0.016 | Not reported | Poor |
| Rubinkiewicz et al, Poland, 2020^43^ | 66 | TATME for low rectal cancer | Initial cases to cases after CUSUM plateau | Increased | Median LOS decreased from 6 to 5 days. p=0.375 | Yes | Poor |
| Dulskas et al, Lithuania, 2015^44^ | 200 | Hand assisted laparoscopic colectomy for left-sided colon and rectal cancer | First 1/2 of cases (100) to remaining cases | Increased | Mean LOS decreased from 8.1 to 6.5 days. p=0.0027 | Not reported | Poor |
| Mackenzie et al, UK, 2016^45^ | 31709 | Laparoscopic colon and rectal cancer surgery | Cases before/after change point | Increased | Median LOS decreased from 8 to 7 days in rectal resection (7 to 6 colon). p<0.001 | Not reported | Poor |
| Tsai et al, Taiwan, 2016^46^ | 240 | Laparoscopic colon and rectal cancer surgery | First 1/3 of cases (80) compared to remaining 2/3 of cases | Increased | Mean LOS decreased from 9.6 in earliest group 8.5 in middle group and 8.8 in latest group. P>0.05 | Not reported | Poor |
| Kim et al, Korea, 2015^47^ | 131 | Single-incision LAR for sigmoid cancer | Cases before/after change point | Increased | Median LOS decreased from 7.1 to 5.5 days. p=0.009 | Not reported | Poor |
| Park et al, Korea, 2015^48^ | 87 | Laparoscopic right hemicolectomy | Cases before/after change point | Increased | Median LOS decreased from 10.7 to 8.4 days. p=0.015 | Not reported | Poor |
| Kirk et al, USA, 2015^49^ | 70 | Single incision laparoscopic right colectomy | First 10 cases to remaining 6 groups of 10 cases each | Increased | Median LOS increased from 6.9 to 7 days. p=0.37 | Not reported | Poor |
| Prakash et al, India, 2013^50^ | 265 | Laparoscopic colon and rectal cancer surgery | First 1/2 of cases (132) to remaining cases | Increased | Mean LOS decreased from 9.3 to 8.6 days. p<0.01 | Not reported | Poor |
| Akmal et al, USA, 2012^51^ | 80 | Robotic TME for rectal cancer (LAR, APR, coloanal) | First 1/2 of cases (40) to remaining cases | Increased | Mean LOS decreased from 7.8 to 7.3 days. p=0.735 | Not reported | Poor |
| Ozturk et al, USA, 2010^52^ | 200 | Hand assisted laparoscopic colon and rectal surgery | First 1/8 of cases (25) to remaining 7/8 of cases | Cannot determine | Median LOS fluctuates between 4 and 5 days but is 4 in both earliest and latest groups. p=0.27 | Yes | Poor |
| Koebrugge et al, Netherlands, 2009^53^ | 105 | Transanal endoscopic microsurgery | First 1/2 of cases (52) to remaining cases | Increased | Mean LOS decreased from 6.1 to 4.4 days. p=0.046 | Not reported | Poor |
| Li et al, HK, China, 2009^54^ | 100 | Laparoscopic colectomy | First 1/2 of cases (50) to remaining cases | Cannot determine | Median LOS 8 days in both groups. p=0.93 | Not reported | Poor |
| Ito et al, Japan, 2009^55^ | 200 | Laparoscopic rectal resection for rectal cancer | First 20 cases to cases 21-40 and subsequent cases | Decreased | Mean LOS 11 days in first 20 cases and 12 days in subsequent cases. p-value not significant, not reported | Not reported | Poor |
| Kim et al, USA, 2007^56^ | 100 | Laparoscopic colon resection performed medial to lateral | First 38 cases (time to improvement in learning curve) to later cases | Cannot determine | Mean LOS 4 days in early and late groups. p-value not significant, not reported | Not reported | Poor |
| Avital et al, Israel, 2006^57^ | 100 | Laparoscopic colon and rectal cancer surgery | First 1/2 of cases (50) to remaining cases | Increased | Mean LOS decreased from 8.6 to 7.2 days. p=0.072 | Not reported | Poor |
| Schlachta et al, Canada, 2001^58^ | 461 | Laparoscopic colon and rectal surgery | First 30 cases to remaining 431 cases | Increased | Median LOS decreased from 6.5 to 5 days. p < 0.001 | Not reported | Poor |

pLOS = prolonged LOS, RC = regression coefficient, OR = odds ratio, RR= risk ratio, HR = hazard ratio, ERAS = Study performed on patients enrolled in a fast-track surgery protocol, NOS = Newcastle Ottawa Scale

**Table S4: Results of studies evaluating the impact of *trainee involvement* on LOS after CRS**

| **Authors, Country, Year, RefID** | **Number of Patients** | **Colorectal Surgeries Included** | **Exposure** | **Impact on LOS** | **Effect Estimate** | **ERAS** | **Risk of Bias/Quality (NOS)** |
| --- | --- | --- | --- | --- | --- | --- | --- |
| Gorgun et al. USA, 2014^60^ | 7254 | Laparoscopic colorectal surgery | Cases with resident participation compared to without resident participation | Increased | Mean LOS 5.77 days in resident group vs. 5.57 days in non-resident group. p=0.028 | Not reported | Good |
| Dalton et al, UK, 2010^61^ | 300 | Laparoscopic colorectal surgery | Cases performed entirely by fellow compared to cases partially by consultant | Increased | Mean LOS 4.6 days in fellow group vs 4.46 days in consultant group. p-value not reported, not significant per author | Not reported | Good |
| Borowski et al, UK, 2007^62^ | 6406 | Colon and rectal cancer surgery | Cases performed by consultants compared to those by supervised trainees | Decreased | Mean LOS 12.4 days for operations by consultants vs 12.0, days for operations by supervised trainees. P=0.002 | Not reported | Good |
| Reddy et al, UK, 2003^63^ | 350 | Colorectal resection | Cases performed by residents compared to cases by consultants | Increased | Regression coefficient -2.25 for surgeries by consultants. p=0.101 | Not reported | Good |
| Homma et al, Japan, 2017^64^ | 78 | Laparoscopic right colectomy | Cases with resident primary surgeon compared to those without resident primary surgeon | Decreased | Mean LOS 1.6 days shorter for patients with resident primary surgeon. p-value not reported, not significant per author | Not reported | Poor |
| Altieri et al, USA, 2016^33^ | 643 | Robotic colectomy | Cases at hospitals with colorectal fellowship programs | Cannot determine | Parameter estimates not provided, no significant relationship between hospitals with colorectal fellowship programs and LOS after CRS. p=0.3855 | Not reported | Poor |
| Celentano et al, UK, 2015^65^ | 151 | Laparoscopic IBD surgery | Cases performed by residents compared to cases with critical steps by consultants | Increased | Mean LOS 8.35 days for cases by supervised trainees and 7.78 for cases by consultants. p=0.6346 | Yes | Poor |
| Krishna et al, Australia, 2013^66^ | 511 | Laparoscopic colorectal surgery | Cases performed by consultants alone compared to those with variable amount of trainee involvement | Increased | Median LOS 8 days for cases by consultants alone and those performed by trainees alone vs. 9 days for those performed by trainees with consultants. p=0.678 | Not reported | Poor |
| Scott Davis et al, USA, 2013^67^ | 89720 | Colectomy | Cases with resident participation compared to without resident participation | Cannot determine | Median LOS 4 days for both groups. Mean not reported. p-value not reported, not significant per author | Not reported | Poor |
| Langhoff et al, Denmark, 2012^68^ | 131 | Colon and rectal cancer surgery | Cases with inexperienced primary surgeon compared to supervisor primary surgeon | Increased | Median LOS 8 days for rectal surgery in both groups, but 1-day shorter median LOS for colon surgery performed by experienced surgeons (4 vs 5 days). p-value not reported, not significant per author | Not reported | Poor |
| Thors et al, USA, 2010^69^ | 159 | Colectomy | Cases with resident participation compared to without resident participation | Decreased | Mean LOS 6.37 for trainee group and 9.95 for non-trainee group. p=0.008 | Not reported | Poor |
| Khan et al, UK, 2008^70^ | 102 | Colon and rectal cancer surgery | Cases performed by trainee primary surgeon compared to cases by consultant | Decreased | Mean LOS 12.1 days for trainee group vs. 14.3 and 14.8 days for consultant and staff grades respectively. p-value not reported, not significant per author | Not reported | Poor |
| Hwang et al, USA, 2008^71^ | 4683 | Colectomy | Cases with resident participation compared to without resident participation | Increased | Mean LOS 12 days with resident involvement and 10.37 days without resident. p-value not reported, not significant per author | Not reported | Poor |
| Mehall et al, USA, 2005^72^ | 451 | Laparoscopic colon and rectal surgery | Cases with compared to without resident participation (PGY 4&5) | Increased | Mean LOS 4.3 days with resident participation and 4.2 days without resident participation. p-value not reported, not significant per author | Not reported | Poor |
| Renwick et al, Australia, 2005^73^ | 494 | Colon and rectal cancer surgery | Cases with resident participation compared to without resident participation | Decreased | Median LOS 11 days at private hospital (no trainee involvement) vs 10 days at public teaching hospital. p<0.001 | Not reported | Poor |

pLOS = prolonged LOS, RC = regression coefficient, OR = odds ratio, RR= risk ratio, HR = hazard ratio, ERAS = Study performed on patients enrolled in a fast-track surgery protocol, NOS = Newcastle Ottawa Scale

**Table S5: Results of studies evaluating the impact of *hospital volume* on LOS after CRS**

| **Authors and Country, RefID** | **Number of Patients** | **Colorectal Surgeries Included** | **Exposure** | **Impact on LOS** | **Effect Estimate** | **ERAS** | **Risk of Bias/ Quality (NOS)** |
| --- | --- | --- | --- | --- | --- | --- | --- |
| Vicendese et al, Australia, 2020^74^ | 28343 | Colorectal cancer surgeries | Hospital volume (annual volume and mean annual volume). Continuous variable | Cannot determine | Increasing LOS with increasing annual volume, but trend reverses at higher annual volumes. Association between mean annual volume and LOS not reported. p-value not reported | Not reported | Good |
| Al-Mazrou et al, USA, 2018^75^ | 9306 | Proctectomy | Lowest tertile of hospitals for proctectomy volume during the study period | Decreased | Adjusted LOS 0.4 days longer for low volume hospitals compared to high volume hospitals. P=0.002 | Not reported | Good |
| Pucciarelli et al, Italy, 2018^76^ | 74307 | Rectal cancer surgery | Highest tertile of hospitals for rectal cancer surgery volume during the study period | Decreased | OR for prolonged LOS 1.54, 2.29 for medium and lowest tertile of hospitals for rectal cancer surgery volume during study period. p-value not reported, significant. | Not reported | Good |
| Lee et al, South Korea, 2017^77^ | 16085 | Colectomy for cancer | Lowest quartile of hospitals for colectomy volume during the study period | Cannot determine | RC (compared to lowest quartile) 0.095, 0.017, -0.061 for two middle and highest quartiles respectively. p-values <0.001, 0.185, <0.001 respectively | Not reported | Good |
| Liu et al, Taiwan, 2015^11^ | 61728 | Colorectal cancer surgeries | Lowest quartile of hospitals for colectomy volume during the study period | Increased | Compared to lowest quartile, OR for pLOS 1.31, 0.86, 0.91 for two middle and highest quartiles respectively. p-values 0.001, 0.165, 0.353 respectively | Not reported | Good |
| Damle et al, USA, 2014^14^ | 17,749 | Colorectal cancer surgeries | Lowest quartile of hospitals for CRS volume | Increased | RC for LOS 0.48, 0.39, 0.18 for second, third, and highest quartiles respectively compared to lowest hospital volume quartile. p-value not reported, not significant. | Not reported | Good |
| Zheng et al, USA, 2014^38^ | 4617 | Laparoscopic colectomy for colon cancer | Hospital greater than 75%ile for laparoscopic colectomy for cancer | Decreased | IRR for LOS 0.94 for high volume sites compared to other sites. p-value not significant. | Not reported | Good |
| Burns et al, UK, 2013^13^ | 109,261 | Colorectal cancer surgeries | Lowest tertile of hospitals for volume AND hospital volume as a continuous variable | Decreased | OR 0.98 and 0.96 for pLOS when middle and highest tertiles respectively compared to lowest tertile of hospitals for volume. p-value not reported, significant. | Not reported | Good |
| Burns et al, UK, 2011^27^ | 5771 | Restorative proctocolectomy (IPAA) | Hospitals in the lowest tertile for aggregated volume during the study period | Decreased | RR 0.93 for highest tertile compared to lowest tertile for volume. p<0.001 | Not reported | Good |
| Faiz et al, UK, 2011^78^ | 186013 | Colon and rectal resections | Lowest tertile of hospitals for colon and rectal surgery volume | Decreased | OR for pLOS 0.88 and 0.89 for medium and high-volume hospitals respectively. p<0.001 | Not reported | Good |
| Kuwabara et al, Japan, 2009^79^ | 3765 | Laparoscopic colectomy | Hospitals performing greater than five laparoscopic colectomy/month during 2007, compared other hospitals in cohort (3 other groupings) | Cannot determine | Compared to highest volume stratum, reduced LOS in lowest 2 strata (RC 0.2, 1.4) but increased LOS in second highest volume stratum (-1.6). p-values 0.723, 0.004 and 0.001 respectively | Not reported | Good |
| Kennedy et al, Canada, 2006^80^ | 1270 | IPAA | High IPAA volume (>100 procedures) compared to medium 10-100) and low (<10) IPAA volume | Increased | Compared to high volume, RC -0.09 and -0.06 for medium and low volume hospitals respectively. Comparison between medium and high strata significant | Not reported | Good |
| Yasunaga et al, Japan, 2009^30^ | 1212 | Laparoscopic colon and rectal resections | Hospitals performing <9 laparoscopic CRS, compared to those performing 10-19, 20-29, 30-39 and >40 laparoscopic CRS | Decreased | HR 1.09 to 2.03 increasing linearly as volume quintile increases (compared to lowest quintile). p<0.01 | Not reported | Fair |
| Keller et al, USA, 2013^34^ | 1428 | Robotic colectomy | Lowest tertile of hospitals for volume | Decreased | Low hospital volume associated with increased LOS (6.99 vs. 5.67). p=0.0053 | Not reported | Poor |
| Kelly et al, Ireland, 2012^35^ | 5133 | Colorectal cancer surgery | Third sextile of hospitals for colorectal cancer surgery volume during the study period | Cannot determine | OR’s for pLOS greater and less than 1for sextiles of higher and lower volume than third (reference) sextile. p-values not reported, only significant relationship between sextiles 3&4 | Not reported | Poor |
| Drolet et al, Canada, 2011^36^ | 54000 | Colectomy for colon cancer | Lowest tertile of hospitals for CRS volume during study period | Increased | 3% increase in LOS on adjusted analysis for medium and high-volume hospitals compared to low volume hospitals. p-value not reported, not significant. | Not reported | Poor |
| Kuhry et al, Netherlands, 2005^81^ | 536 | Laparoscopic colectomy for colon cancer | Lowest tertile of hospitals for laparoscopic colectomy volume during the study period | Decreased | Median LOS 6, 7 and 8 days for high, medium and low volume hospitals respectively. p<0.001 | Not reported | Poor |

pLOS = prolonged LOS, RC = regression coefficient, OR = odds ratio, RR= risk ratio, HR = hazard ratio, ERAS = Study performed on patients enrolled in a fast-track surgery protocol, NOS = Newcastle Ottawa Scale

**Table S6: Results of studies evaluating the impact of *teaching hospital status* on LOS after CRS**

| **Authors and Country, RefID** | **Number of Patients** | **Colorectal Surgeries Included** | **Exposure** | **Impact on LOS** | **Effect Estimate** | **ERAS** | **Risk of Bias/ Quality (NOS)** |
| --- | --- | --- | --- | --- | --- | --- | --- |
| Dowzicky et al, USA, 2019^82^ | 32732 | Colorectal surgeries | Teaching hospital compared to non-teaching hospitals | Increased | OR for LOS < 25^th^ percentile 0.96 for teaching hospitals. p-value not significant | Not reported | Good |
| Cagino et al, USA, 2018^83^ | 12517 and 3996 respectively | Low anterior resection (LAR) for rectal cancer and with stoma formation | Hospitals associated with general surgery residency program or medical school compared to other hospitals in cohort | Increased | LOS ratio for academic vs. community hospitals 1.05 (significant) and 1.06 (p=0.0433) for LAR and LAR with stoma formation respectively. | Not reported | Good |
| Zheng et al, USA, 2014^38^ | 4617 | Laparoscopic colectomy for colon cancer | Hospitals with medical school affiliations compared to those without | Decreased | Incident RR for LOS 0.89 for hospitals with medical school affiliations. p-value significant. | Not reported | Good |
| Rea et al, USA, 2011^26^ | 113,633 | Colon and rectal resections | Urban non-teaching hospitals compared to urban teaching hospitals | Increased | RC -0.32 for urban non-teaching hospitals compared to urban teaching hospitals. p<0.0001 | Not reported | Good |
| Hayanga et al, USA, 2010^84^ | 115250 | Colectomy | Teaching hospitals compared to non-teaching hospitals | Increased | Mean LOS 0.52 days longer in teaching hospitals after adjustment. p=0.003 | Not reported | Good |
| Kuwabara et al, Japan, 2009^79^ | 3765 | Laparoscopic colectomy | Academic hospitals compared to community hospitals | Decreased | RC for LOS -0.3 for academic hospitals compared to community hospitals. p=0.448 | Not reported | Good |
| Freischlag et al, USA, 2019^85^ | 80922 | MIS segmental colectomy for colon cancer | Academic hospitals (associated with medial schools or designated National Cancer Institute Comprehensive Cancer Care Programs). Compared to comprehensive (>650 annual cases) and community hospitals (100-650 annual cases) | Decreased | Mean LOS 5.99 days in academic hospitals compared to 6.00 and 6.18 days in comprehensive and community hospitals respectively. p<0.0001 | Not reported | Poor |
| Khuri et al, USA, 2001^86^ | 9329 | Colectomy | Hospital with Dean’s committee and at least one surgical resident compared to other hospitals in cohort | Increased | Mean LOS 12.1 days in academic hospitals compared to 11.2 in other hospitals. p<0.0001 | Not reported | Poor |

pLOS = prolonged LOS, RC = regression coefficient, OR = odds ratio, RR= risk ratio, HR = hazard ratio, ERAS = Study performed on patients enrolled in a fast-track surgery protocol, NOS = Newcastle Ottawa Scale

**Table S7: Results of studies evaluating the impact of *hospital rurality* on LOS after CRS**

| **Authors and Country, RefID** | **Number of Patients** | **Colorectal Surgeries Included** | **Exposure** | **Impact on LOS** | **Effect Estimate** | **ERAS** | **Risk of Bias/ Quality (NOS)** |
| --- | --- | --- | --- | --- | --- | --- | --- |
| Udayasiri et al, Australia, 2020^87^ | 18470 | Colorectal cancer surgery (major) | Metropolitan hospitals compared to inner and outer regional hospitals | Decreased | OR for pLOS 0.53 and 0.64 for inner and outer regional hospitals respectively. p<0.001 | Not reported | Good |
| Aquina et al, USA, 2016^12^ | 6295 | Reversal of Hartmann’s colostomy | Urban hospitals compared to rural hospitals | Decreased | Adjusted incident RR 1.09 for urban hospitals compared to rural hospitals. p-value not reported, not significant. | Not reported | Good |
| Pandit et al, USA, 2016^88^ | 20617 | Benign colorectal surgery | Rural hospitals compared to urban hospitals | Increased | Mean LOS 4.7 days in urban hospitals compared to 5.9 days in rural hospitals. p<0.001 | Not reported | Poor |

pLOS = prolonged LOS, RC = regression coefficient, OR = odds ratio, RR= risk ratio, HR = hazard ratio, ERAS = Study performed on patients enrolled in a fast-track surgery protocol, NOS = Newcastle Ottawa Scale

**Table S8: Results of studies evaluating the impact of *private hospitals* on LOS after CRS**

| **Authors and Country, RefID** | **Number of Patients** | **Colorectal Surgeries Included** | **Exposure** | **Impact on LOS** | **Effect Estimate** | **ERAS** | **Risk of Bias/ Quality (NOS)** |
| --- | --- | --- | --- | --- | --- | --- | --- |
| Vicendese et al, Australia, 2020^74^ | 28343 | Colorectal cancer surgeries | Surgery at public hospitals compared to surgery at private for-profit hospitals | Cannot determine | For low LOS percentiles, longer LOS in private hospitals. No significant relationship for middle percentiles. For high LOS percentiles, longer LOS at public hospitals. p-value not reported | Not reported | Good |
| Liu et al, Taiwan, 2015^11^ | 61,728 | Colorectal cancer surgeries | Surgeries at public hospitals compared to surgeries at private hospitals | Decreased | OR for pLOS 1.15 for public hospitals. p<0.023 | Not reported | Good |
| Zheng et al, USA, 2014^38^ | 4617 | Laparoscopic colectomy for colon cancer | Surgery at government owned hospitals compared to privately owned hospital | Increased | IRR for LOS after surgery at government hospitals 0.96 compared to private hospitals. p-value not reported, not significant. | Not reported | Good |
| Rickard et al, Australia, 2004^89^ | 1095 | Colorectal cancer surgery | Surgeries at private hospitals compared to surgery at public hospitals | Increased | Regression coefficient 1.4 for LOS after surgeries in private hospitals. p=0.0152 | Not reported | Good |
| Renwick et al, Australia, 2005^73^ | 494 | Colon and rectal cancer surgery | Surgeries at public hospitals compared to surgeries at private hospitals | Increased | Median LOS 11 days at private hospital vs 10 days at public hospital. p<0.001 | Not reported | Poor |

pLOS = prolonged LOS, RC = regression coefficient, OR = odds ratio, RR= risk ratio, HR = hazard ratio, ERAS = Study performed on patients enrolled in a fast-track surgery protocol, NOS = Newcastle Ottawa Scale
