# Supplementary figures and images for: Impact of surgeon and hospital factors on length of stay after colorectal surgery systematic review
Source: BJS Open. 2022 Sep 19;6(5):zrac110. doi: 10.1093/bjsopen/zrac110 (PMC9487584; doi:10.1093/bjsopen/zrac110)

**Appendix S1: PRISMA Checklist**


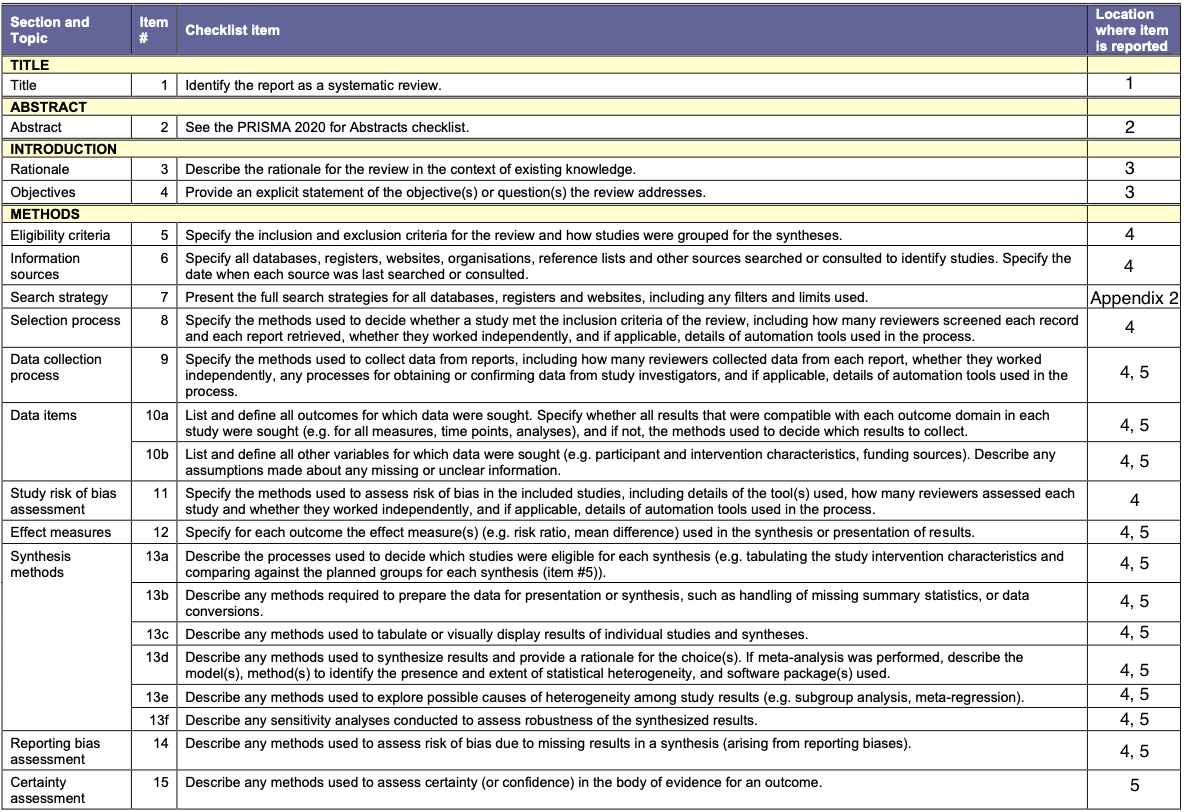


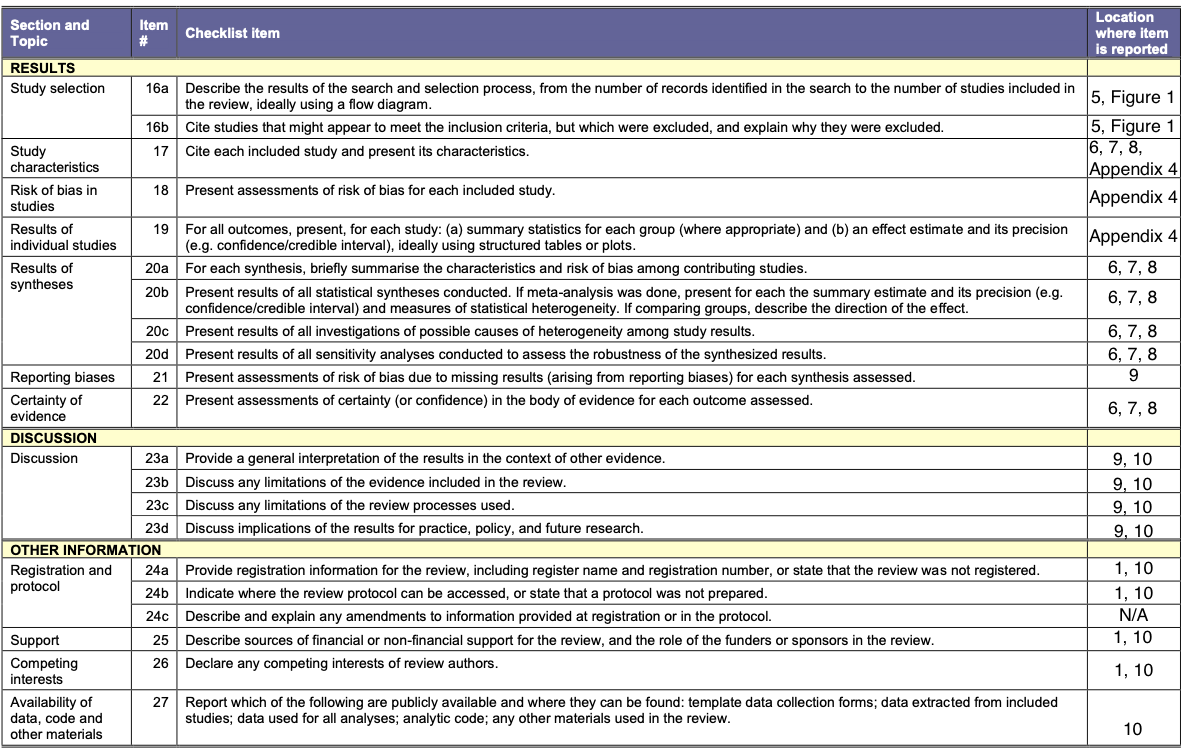


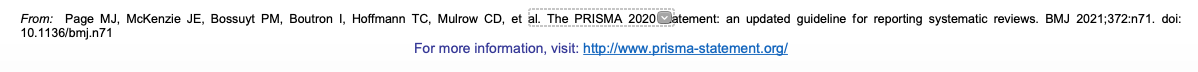

Supplement: zrac110_Supplementary_Data [file zrac110_supplementary_data.zip › Supplementary_Appendix_1.docx]
